# Identification of Diterpenoid Alkaloids from the Rootsof *Aconitum kusnezoffii* Reihcb

**DOI:** 10.3390/molecules16043345

**Published:** 2011-04-19

**Authors:** Ning Xu, De-Feng Zhao, Xin-Miao Liang, Hua Zhang, Yuan-Sheng Xiao

**Affiliations:** 1 School of Chemical Engineering, Dalian University of Technology, Dalian 116000, China; 2 Key Lab of Separation Science for Analytical Chemistry, Dalian Institute of Chemical Physics, Chinese Academy of Sciences, Dalian 116023, China

**Keywords:** roots of *Aconitum kusnezoffii Reichb.*, diterpenoid alkaloid, aconitum

## Abstract

Three diterpenoid alkaloids, including an unreported compound, were isolated from the roots of *Aconitum kusnezoffii* Reichb. On the basis of spectral analysis, these three compounds were determined to be 1,15-dimethoxy-3-hydroxy-14-benzoyl-16-ketoneoline, benzoylaconine and aconitine.

## 1. Introduction

*Aconitum kusnezoffii* Reichb., belonging to the genus *Aconitum L.*, is distributed in the Xinjiang, Sichuan and Jilin provinces of China. Although recently there were some reports on flowers [1] and leaves [2] of this plant, roots of *Aconitum kusnezoffii* Reichb. had been long used in Traditional Chinese Medicine as an analgesic and cardiotonic herbal medicine. Diterpenoid alkaloids [3,4] and polysaccharide [5] have been isolated from its roots to date. To our knowledge, diterpenoid alkaloids extracted from the genus *Aconitum L.* have various pharmacological properties [6,7,8,9,10]. In the search for biologically active alkaloids from the roots of *Aconitum kusnezoffii* Reichb., a detailed study was carried out. This led to the isolation of the new diterpenoid alkaloid 1,15-dimethoxy-3-hydroxy-14-benzoyl-16-ketoneoline, along with two known diterpenoid alkaloids, benzoylaconine and aconitine. Herein, the structure of the new compound was determined based on MS, IR, and NMR spectral data, and the known ones were identified by comparing their NMR data with those in the literature [3].

## 2. Results and Discussion

Compound **1** was obtained as a white solid. Its molecular formula was determined to be C_32_H_4__3_O_9_N by ESI-MS (M/Z 586. 861 [M+H]^+^). The ^1^H-NMR spectroscopic data of compound **1** showed three groups of absorptions for a benzoyl moity at ***δ*_H_** 7.47 (2H, t, H-4ʺ), 7.61 (1H, t, H-5ʺ), and 7.96 (2H, d, H-3ʺ); N-ethyl protons at ***δ*_H_** 2.72 (1H, m, H-1ʹα), 2.74 (1H, m, H-1ʹβ), and 1.14 (3H, t, H-2ʹ); and four *O*-methyl protons at ***δ*_H_** 3.28 (3H, s, CH_3_O-1) 3.29 (3H, s, CH_3_O-6), 3.30 (3H, s ,CH_3_O-18), and 3.82 (3H, s, CH_3_O-15). The ^13^C-NMR and DEPT spectra of **1** revealed the presence of 32 carbons, which were assignable to a tertiary methyl group at ***δ*_C_** 12.2 (C-2), an oxygenated methylene group at ***δ*_C_** 76.3 (C-18), an oxygenated quaternary carbon at ***δ*_C_** 77.5 (C-8), two quaternary carbons at ***δ*_C_** 43.6 (C-4) and 51.2 (C-11), four methylene groups at ***δ*_C_** 31.8 (C-12), 32.6 (C-2), 48.5 (C-1ʹ) and 49.3 (C-19), two carbonyl groups at ***δ*_C_** 166.0 (C-1ʺ) and 211.8 (C-16) and aromatic hydrocarbon signals at ***δ*_C_** 128.7 (C-4ʺ), 129.1 (C-2ʺ), 129.7 (C-3ʺ) and 133.8 (C-5ʺ), respectively. After further analysis of the spectroscopic data, compound **1** was identified as a neoline-type diterpenoid alkaloid [11].

**Figure 1:**
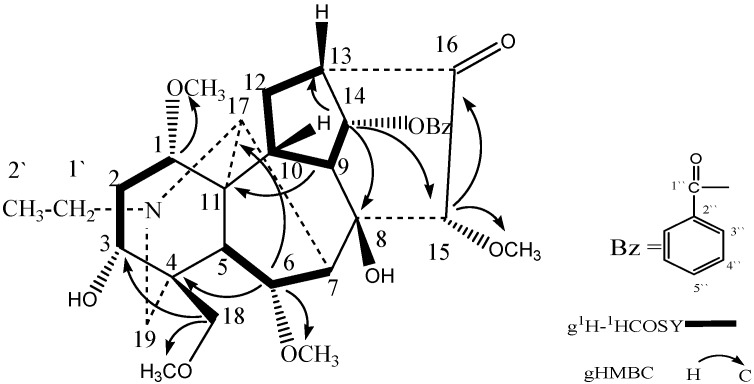
Structure and Key Correlations of Compound **1**.

The g^1^H–^1^H COSY, gHSQC and gHMBC spectra allowed the complete assignment of the chemical shifts of compound **1** (Table 1) [12,13,14,15]. The ^1^H-NMR and ^13^C-NMR resonances of compound **1** were similar to those of chasmanine [3], which was also isolated from roots of *Aconitum kusnezoffii* Reichb., except for the different configurations of C-3, C-15, C-16 and C-14. The main differences are that the chemical shifts of C-3, C-15, C-16 and H-14 are ***δ*_C_** 71.0, 85.8, 211.8 and ***δ*_H_** 5.44 (d, *J* = 5.0 Hz) respectively in compound **1**, and ***δ*_C_** 35.0, 38.7, 81.9 and ***δ*_H_** 4.12 (t, *J* = 4.5 Hz) respectively in chasmanine, which suggested that C-3, C-15 and C-16 in compound **1** belong to an oxygen-containing group. In the gHMBC spectrum, the correlations between H-18 and C-3, and between H-15 and CH_3_O-15 were important to confirm the presence of oxygen groups at C-3 and C-15. Further analysis of the ^13^C NMR data suggested that C-16 in compound **1** could be a carbonyl group, and not methoxyl group. The gHMBC correlations from C-1ʺ to H-14 and H-3ʺ confirmed the position of a benzoyl group. In addition, it is noteworthy that the chemical shift of H-14 in compound **1** was more deshielded than the same proton in chasmanine as the result of the steric effect of the carbonyl group at C-16. On the basis of the above spectral data, compound **1** was determined to be 1,15-dimethoxy-3-hydroxy-14-benzoyl-16-ketoneoline (Figure 1).

**Table 1:** 1D and 2D NMR Data for Compound **1** in CDCl_3_.

	*δ*_H_	*δ*_C_	g^1^H_^1^HCOSY	gHSQC
1	3.20 m	82.5	H-2α, H-2β	+
2α	1.97 m	32.6	H-1, H-2β, H-3	+
2β	2.38 m		H-1, H-2α, H-3	+
3	3.88 d *J* = 4.9 Hz	71.0	H-2α, H-2β	+
4	-	43.6	-	-
5	2.23 d *J* = 6.4 Hz	46.5	H-6, H-7, H-17	+
6	3.95 d *J* = 6.6 Hz	83.7	H-5, H-7, H-17	+
7	2.79 m	42.3	H-6	+
8	-	77.5	-	-
9	2.84 m	38.0	H-10, H-14	+
10	2.33 m	44.2	H-9, H-12α, H-12β	+
11	-	51.2	-	-
12α	1.92 m	31.8	H-10, H-12β, H-13	+
12β	2.91 dd *J* = 15.3 Hz *J* = 6.9 Hz		H-10, H-12α	+
13	2.67 m	49.4	H-12α	+
14	5.44 d *J* = 5.0 Hz	78.3	H-9	+
15	3.86 s	85.8	-	+
16	-	211.8	-	-
17	3.15 s	62.1	H-5, H-6	+
18α	3.59 d *J* = 8.7 Hz	76.3	H-18β	+
18β	3.70 d *J* = 8.7 Hz		H-18α	+
19α	2.65 d *J* = 11.9 Hz	49.3	H-19β	+
19β	3.25 d *J* = 11.9 Hz		H-19α	+
CH_3_O-1	3.28 s	55.9	-	+
CH_3_O-6	3.29 s	58.3	-	+
CH_3_O-15	3.82 s	62.4	-	+
CH_3_O-18	3.30 s	59.2	-	+
N-CH_2_ 1ʹα	2.72 m	48.5	H-2ʹ	+
1ʹβ	2.74 m		H-1α, H-2ʹ	+
-CH_3_ 2ʹ	1.14 t *J* = 7.0 Hz	12.2	H-1ʹα, H-1ʹβ	+
Bz				
1ʺ	-	166.0	-	-
2ʺ	-	129.1	-	-
3ʺ	7.96 d *J* = 7.6 Hz	129.7	H-4ʺ	+
4ʺ	7.47 t *J* = 7.5 Hz	128.7	H-3ʺ, H-5ʺ	+
5ʺ	7.61 t *J* = 7.1 Hz	133.8	H-4ʺ	+

## 3. Experimental

### 3.1. General

IR spectra were recorded using a Thermo Nicolet Nexus spectrophotometer. 1D and 2D NMR spectra were obtained on Bruker AV400; ***δ*_H_** values are expressed in parts per million relative to the solvent (CDCl_3_) signal. DEPT, g^1^H-^1^H COSY, gHSQC, and gHMBC experiments were carried out with the pulse sequences given by Bruker. LC-MS were performed on a UPLC instrument (Waters, USA) coupled with a ZQ 2000 mass spectrometer (Waters, USA) using an XTerra MS C_18_ 2.1 × 150 mm column (Waters, USA) and a C_18_HCE 4.6 × 150 mm column (self-made). A Waters Auto-Purification Factory was used in this study and consisted of a sample inJector (Waters 2777 sample manager), a passive splitter, a compensation pump (515 HPLC pump), an eight-channel UV detector (MUX-UV 2488), a four-channel MS detector (Micromass ZQ2000), four Waters 2525 binary gradient modules, and four Waters 2757 sample managers using XTerra MS C_18_ 19 × 150 mm (Waters, USA) and C_18_HCE 10 × 150 mm (self-made) columns. Data were collected using a MassLynx workstation.

### 3.2. Plant Material

Roots of *Aconitum kusnezoffii* Reichb. were collected in YiLi, Xinjiang Province, China, in July 2008. The herb was authenticated by Xi Rong He, Institute of Medication, Xiyuan Hospital of China Academy of Traditional Chinese Medicine. A voucher specimen (CAOWU 200807) was deposited at the School of Chemical Engineering, Dalian University of Technology.

### 3.3. Extraction and Isolation

Dried powdered roots of the plant (50.0 kg) were defatted with EtOH (500.0 L) over 12 h. After removing the solvent under vacuum at 45 ºC, the residue was applied to X-4 macroporous absorption resin and eluted with H_2_O followed in sequence by 30%, 50% and 100% EtOH (v/v, aqueous-EtOH). The EtOH fractions were separated by column chromatography on silica gel eluted with petroleum, petroleum/EtOAc (v/v, 5:1), EtOAc and EtOH to obtain four fractions. Fraction 4 (15.0 g) was treated with a Waters Auto-Purification Factory using an XTerra 19 × 150 mm column. The injection volume was 1.2 mL. The flow rate was 17 mL/min. The mobile phases were 30 mM ammonium acetate buffer (pH = 8.0) and acetonitrile. Linear gradient elution was adopted starting from 10% Bp to 90% Bp: 0–40 min, 10–50% Bp; 40–45 min, 50–90% Bp and 45–60 min, 90–90% Bp. Fraction 4 was separated to afford 40 fractions of 1.5 min each. Fractions 16-17 were separated by Pre-HPLC with formic acid/water (0.2:100, v/v) and acetonitrile on C_18_HCE 10 × 150 mm. The injection volume was 1.0 mL. The flow rate was 3 mL/min. Linear gradient elution was as follows: 0–10 min, 5–20% Bp; 10–30 min, 20–25% Bp and 30–50 min, 25–50% Bp. Fractions of 1 min each were collected. This separation yielded 1,15-dimethoxy-3-hydroxy-14-benzoyl-16-ketoneoline (4.1 mg), benzoylaconine (5.2 mg) and aconitine (3.2 mg).

### 3.4. Spectral Data

1,15-Dimethoxy-3-hydroxy-14-benzoyl-16-ketoneoline: amorphous solid; IR (KBr) *v*_max_ 3480 (OH), 2935 (CH), 1780 (C=O, ester), 1451, 1382, 1320, 1282, 1180, 1,097, 983, 711 cm^-1^; UV λ*_max_* (CAN/H_2_O) 232 nm; ^1^H- and ^13^C-NMR data, Table 1; ESI-MS M/Z 586.861 [M+H]^+^ (calcd for C_32_H_4__3_O_9_N, 585. 041).

## 4. Conclusions

Based on the results of our present study, roots of *Aconitum kusnezoffii* Reichb. contain diterpenoid alkaloids, which possess diverse biological properties, as the principal secondary metabolites. A new diterpenoid alkaloid, 1,15-dimethoxy-3-hydroxy-14-benzoyl-16-ketoneoline, together with two known alkaloids, benzoylaconine and aconitine, were separated from roots of *Aconitum kusnezoffii* Reichb., and their structures identified by spectral analysis.

## Supplementary Materials

Supplementary materials can be accessed at  http://www.mdpi.com/1420-3049/16/4/3345/s1.
