# Supplementary material for: Identification of Diterpenoid Alkaloids from the Rootsof Aconitum kusnezoffii Reihcb
Source: Molecules. 2011 Apr 19;16(4):3345–50. doi: 10.3390/molecules16043345 (PMC6260597; doi:10.3390/molecules16043345)

Supporting information

-**S1**: ^{1}H-NMR Spectrum of Compound 1 in CDCl3

-**S2**: ^{13}C-NMR Spectrum of Compound 1 in CDCl3

- **S3**: DEPT Spectrum of Compound 1 in CDCl3

- **S4**: g^{1}H-^{1}H COSY Spectrum of Compound 1 in CDCl3

- **S5**: gHSQC Spectrum of Compound 1 in CDCl3

- **S6**: gHMBC Spectrum of Compound 1 in CDCl3

S1: ^{1}H-NMR Spectrum of Compound 1 in CDCl3

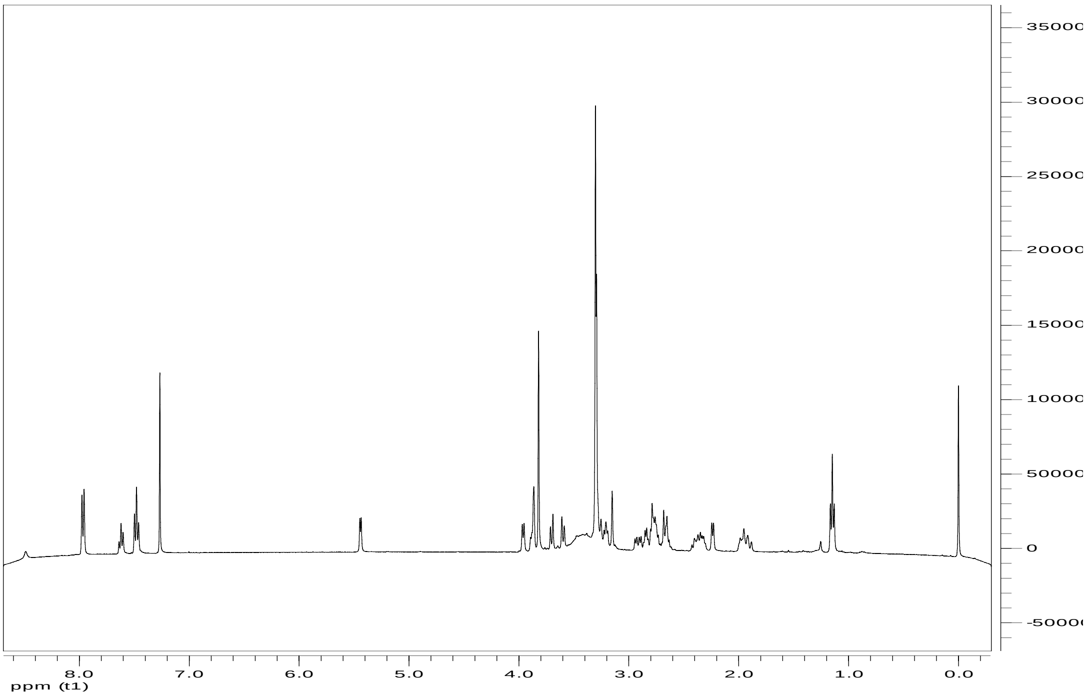

S2: ^{13}C-NMR Spectrum of Compound 1 in CDCl3

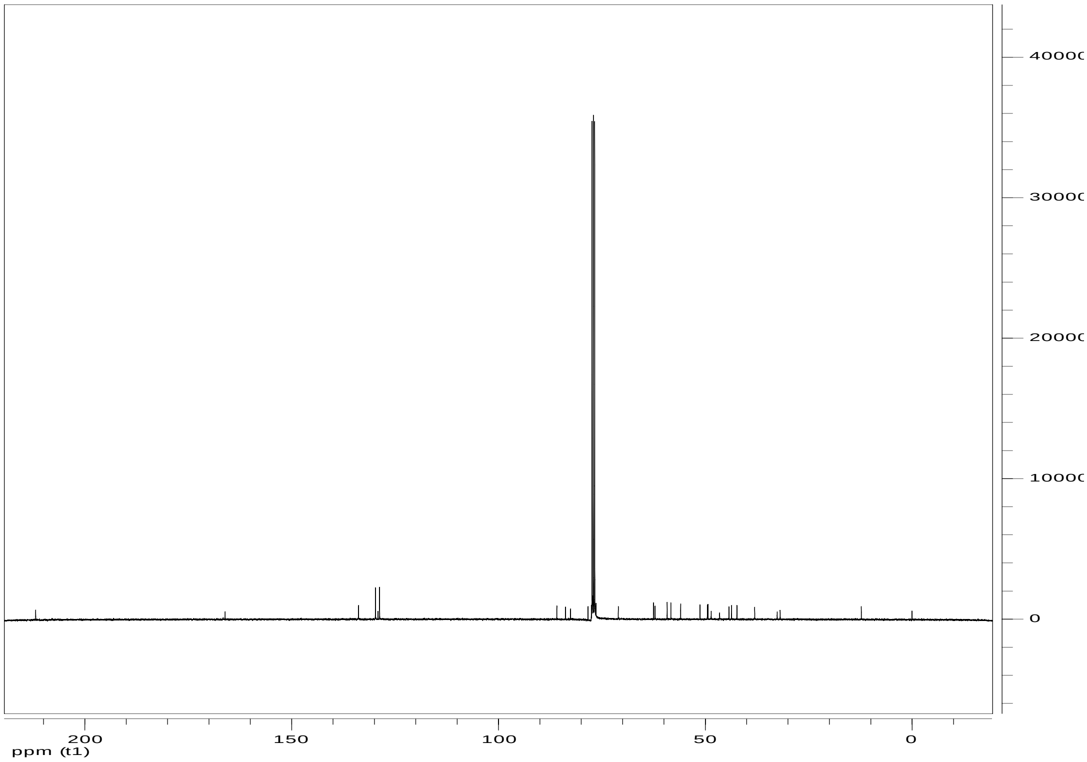

S3: ^{13}C DEPT Spectrum of Compound 1 in CDCl3

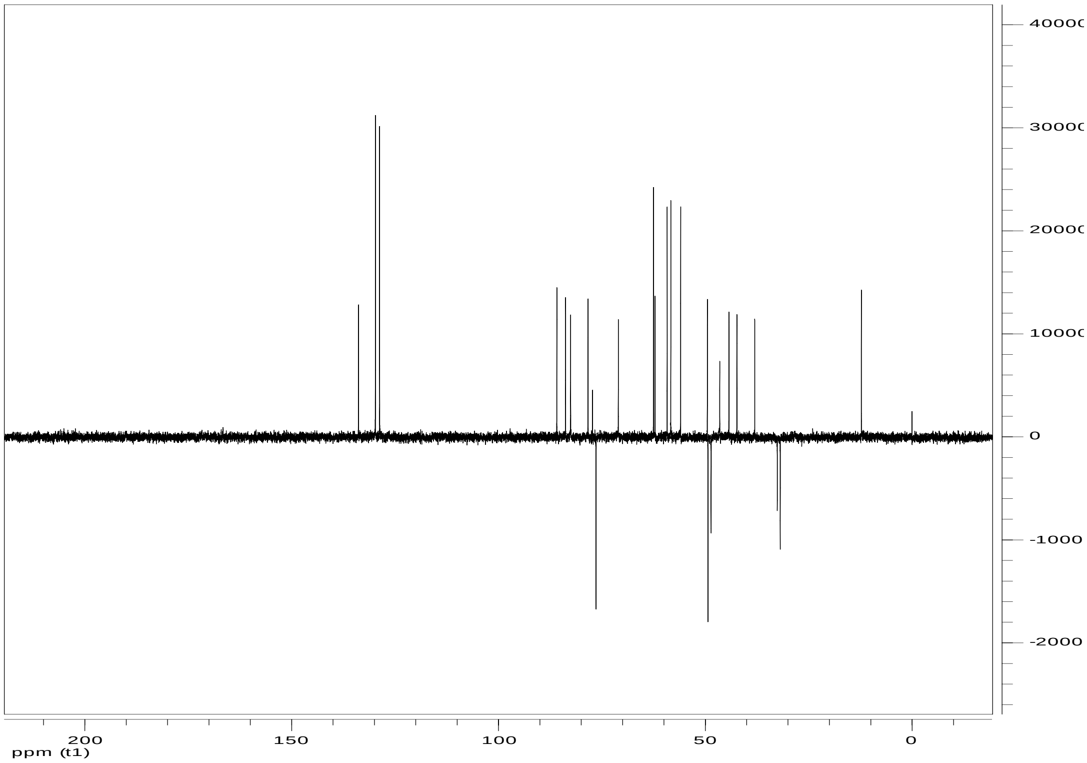

S4: g^{1}H-^{1}HCOSY Spectrum of Compound 1 in CDCl3

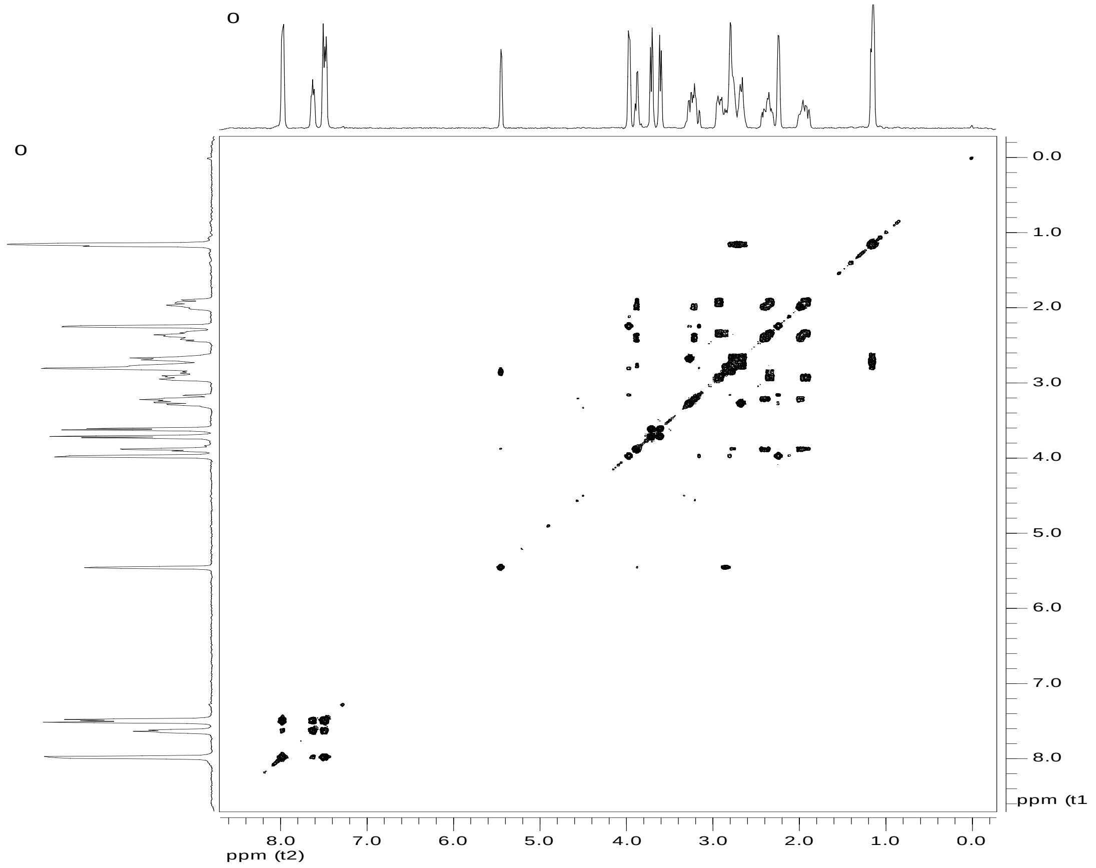

S5: gHSQC Spectrum of Compound 1 in CDCl3

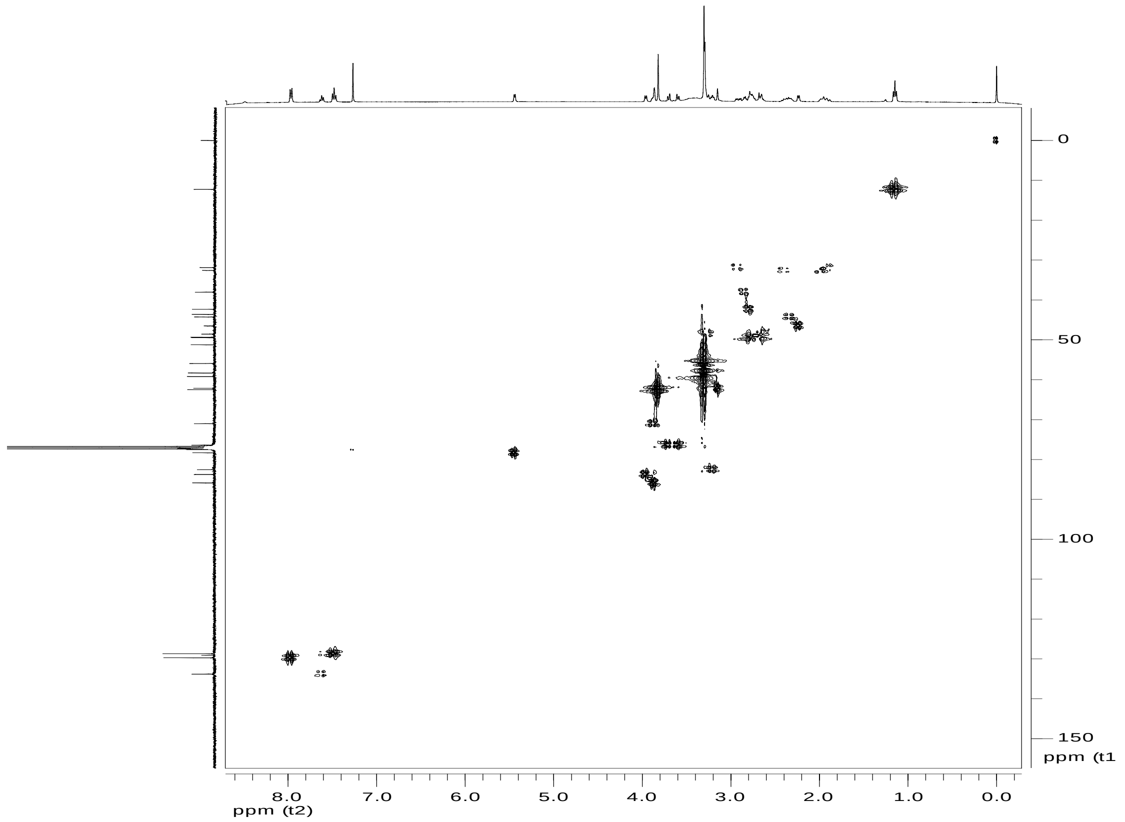

S6: gHMBC Spectrum of Compound 1 in CDCl3

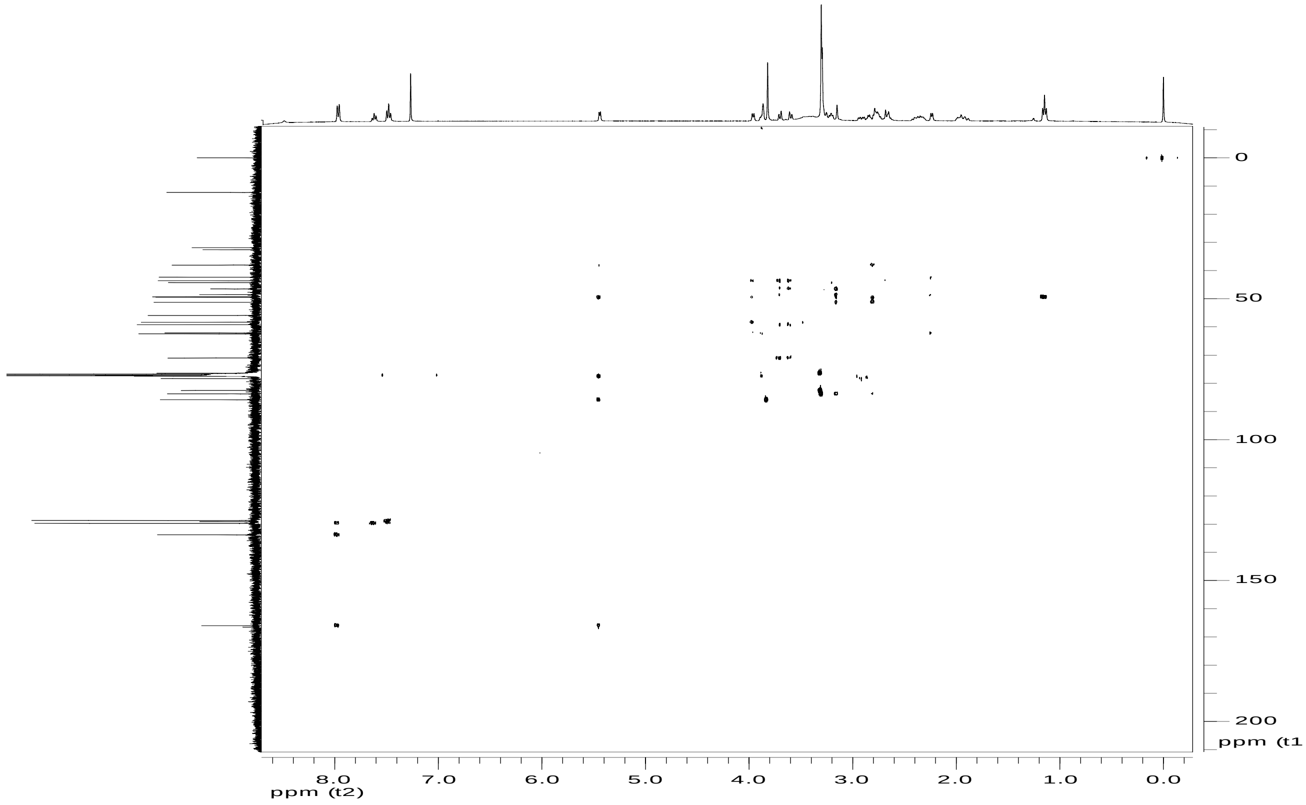

Supplement: Supplementary file 1 [file molecules-16-03345-s001.pdf]
